# Microalgae as a Source of Mycosporine-like Amino Acids (MAAs); Advances and Future Prospects

**DOI:** 10.3390/ijerph182312402

**Published:** 2021-11-25

**Authors:** Subhisha Raj, Anusree M. Kuniyil, Arathi Sreenikethanam, Poornachandar Gugulothu, Rajesh Banu Jeyakumar, Amit K. Bajhaiya

**Affiliations:** 1 Algal Biotechnology Lab, Department of Microbiology, Central University of Tamil Nadu, Thiruvarur 610104, Tamil Nadu, India; subhisharaj1997@gmail.com (S.R.); anusreebinduanu@gmail.com (A.M.K.); arathi0517@gmail.com (A.S.); 2 Department of Life Sciences, Central University of Tamil Nadu, Thiruvarur 610104, Tamil Nadu, India; poornachandar@cutn.ac.in (P.G.); rajeshbanu@cutn.ac.in (R.B.J.)

**Keywords:** mycosporine-like amino acids, microalgae, photoprotective activity, anti-cancer activity, genetic engineering, bio-refinery

## Abstract

Mycosporine-like amino acids (MAAs), are secondary metabolites, first reported in 1960 and found to be associated with the light-stimulated sporulation in terrestrial fungi. MAAs are nitrogenous, low molecular weight, water soluble compounds, which are highly stable with cyclohexenone or cycloheximine rings to store the free radicals. Microalgae are considered as a good source of different kinds of MAAs, which in turn, has its own applications in various industries due to its UV absorbing, anti-oxidant and therapeutic properties. Microalgae can be easily cultivated and requires a very short generation time, which makes them environment friendly source of biomolecules such as mycosporine-like amino acids. Modifying the cultural conditions along withmanipulation of genes associated with mycosporine-like amino acids biosynthesis can help to enhance MAAs synthesis and, in turn, can make microalgae suitable bio-refinery for large scale MAAs production. This review focuses on properties and therapeutic applications of mycosporine like amino acids derived from microalgae. Further attention is drawn on various culture and genetic engineering approaches to enhance the MAAs production in microalgae.

## 1. Introduction

Secondary metabolites are specialized natural products which are not directly involved in general metabolism of an organism, but they have specialized functions such as antibiotic, pharmacological, photoprotective, anti-inflammatory activities, etc. [1]. Several organisms which belong to classes like microalgae, macroalgae and fungi, when exposed to high light intensities can produce a class of secondary metabolite called mycosporine-like amino acids (MAAs), which are also known as microbial sunscreen. MAAs were first reported in terrestrial fungi and the first description of the compound was done in cyanobacteria *Gloeocapsa* sp. strain under high UV radiation [2]. The molecular weight of MAAs varies from 188 Da to 1050 Da [3].

MAAs play a vital role in maintaining the osmotic equilibrium of many organisms especially in photosymbiotic partnerships. The structure of the first mycosporine-like amino acid (MAA) isolated from zoanthid *Palythoa tuberculosa* is of mycosporine-glycine, which was established by Ito and Hirata [4]. The structure has a chromophore ring with amino alcohol at C1 position and a glycine moiety at C3 position. They are highly stable compounds with the resonance structures and zwitterion formation [5].

The two biosynthesis pathways of MAAs include the shikimate pathway and pentose phosphate pathway, but most of the produced MAAs come through the shikimate pathway. MAAs are well known for their UV (Ultra violet) rays absorbing properties, where these compounds can accommodate their free radicals in their core ring structure [6]. Hence, MAAs are considered as free radical scavengers, which can take up the ROS (Reactive oxygen species) to reduce cell damage produced during oxidative stress [7]. MAAs can act as anticancer agents either by controlling proliferation of cells or by stimulating apoptosis in cancerous cells, which leads to the cell death. MAAs are also known to have the ability in stimulating the factors and signals involved in the pathway for the healing of wounds [8].

Many groups of organisms like macroalgae, microalgae, marine heterotrophic bacteria, fungi and various other marine organisms are known to produce mycosporine-like amino acids which are involved in some of their survival processes and stress defense mechanisms. They also act as an intracellular nitrogen reservoir in some species. In aquatic organisms like algae and various marine organisms, these biomolecules are involved in the protection against harmful solar radiation. Specifically in certain microalgae, these MAAs protect them from the inhibition of motility by UVB radiation exposure [9]. Mycosporine-like amino acids are not only known for its photoprotective activities in microalgae, but are also involved in protecting them from salt stress by regulating osmotic balance [10]. In some cyanobacteria species, these molecules have a significant role in protecting the cells from desiccation stress. Also, in certain algal species, they were observed to be involved in intracellular nitrogen storage and photosynthetic activities [11].

Different conditions and parameters were shown to have a significant impact on MAAs expression in certain species of microalgae. In some species like *Alexandrium excavutum*, *Phaeocystis pouchetii*, and the diatom *Guinardia striata*, it was found that there is a strong increase in MAAs content when exposed to sunlight. In certain other species, it was also observed that the accumulation of MAAs in their cells has a strong correlation with the spectral composition of the light [9,11].

It was found that about 152 species of marine microalgae produce MAAs, in which the highest ratio of these compounds was observed in the bloom forming algal groups like dinoflagellates, prymnesiophytes, raphidophytes, and cryptomonads. However, in some cases, irrespective of their taxonomic affiliation, the expression of MAAs molecules in these marine organisms solely depended on their exposure to UV radiation and high intensity light [11]. Table 1 summarizes some of the prominent microalgal and cyanobacterial species, which are known to produce different types of MAAs. 

In addition to the traditional MAAs extraction techniques, identification of genes involved in MAAs biosynthesis and the application of genetic engineering can be utilizedto increase the expression of MAAs in microalgae. Also, various environmental parameters and culture conditions can be modified to increase the accumulation of MAAs in algal cells [27].

## 2. Effect of Culture Conditions on Mycosporine-like Amino Acids Production, and Their Characterization

**Figure 1:**
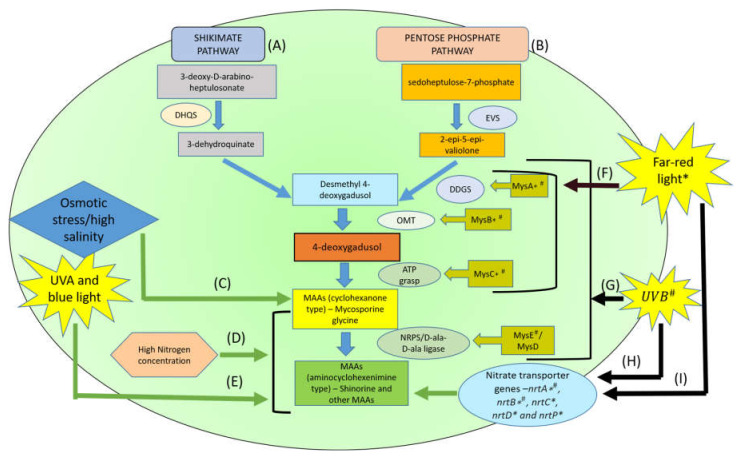
Different abiotic factors affecting the MAAs biosynthesis in microalgae and cyanobacteria. (**A**) Shikimate pathway and (**B**) Pentose phosphate pathway for MAAs biosynthesis [28,33], (**C**) High salinity/osmotic stress induces high MAAs production-particularly mycosporine-glycine production [10,29], (**D**) High nitrogen concentration induces high MAAs production [28,31,32], (**E**) Exposure to UVA and blue light within PAR region of the light spectrum induces high MAAs production [27], (**F**) Exposure to far-red light upregulates MysA, MysB and MysC genes involved in MAAs biosynthesis–increases MAAs production [28], (**G**) Exposure to UVB radiation upregulates MysA, MysB, MysC and MysE genes involved in MAAs biosynthesis–increases MAAs production [28], (**H**) Exposure to UVB rays upregulates nitrate transporter genes nrtA and nrtB in the presence of high nitrogen concentration-increases MAAs production [28], (**I**) Exposure to far-red light upregulates nitrate transporter genes nrtA, nrtB, nrtC, nrtD and nrtP in the presence of high nitrogen concentration-increases MAAs production [28]. DHQS–Dehydroquinate synthase, DDGS- desmethyl-4-deoxygadusol synthase, OMT- O-methyltransferase, EVS-epi-valiolone synthase, NRPS- non-ribosomal peptide synthetase, * Genes upregulated by far-red light, # Genes upregulated by UVB radiation.

It was found that the spectral composition of light in which the algae are exposed have a significant effect on the MAAs content in them. Blue light and UVA radiation was observed to have more effect in increasing the MAAs production compared to red and green light [9]. Study performed by Jose I. Carreto (2001) on dinoflagellates species, *Alexandrium tamarense*, *Alexandrium catenella* and *Alexandrium minutum* showed induced MAAs synthesis on exposure to high light conditions (65 ± 5 µmol quanta m^−2^ s^−1^ PAR) [16]. Similar results were observed on treating cyanobacterium *Microcystis aeruginosa* [17] under continuous high white fluorescent light and on *Scytonema* cf. *crispum* (UCFS10 and UCFS15) under alternative 12-h of day and night cycle [22]. Light conditions/spectra (UV conditions) by which the cultures are exposed can affect the MAAs profiles and their concentration in them. Exposure to UVB radiation and far-red light had a significant impact on MAAs production in cyanobacterium *Chlorogloeopsis fritschii* PCC 6912 [28]. They were responsible for the upregulation of genes (Mys gene cluster) involved in MAAs biosynthesis. They also upregulated nitrate transporter genes (nrtA, nrtB, nrtC, nrtD and nrtP), which increased the MAAs production in presence of increased nitrogen concentration [28]. When freshwater microalga *Desmodesmus* sp. was grown under high saline conditions, it was able to accumulate more MAAs in its cells, which helped it to survive under salt stress [29]. Similar results were also observed in *Aphanothece halophytica*, a halotolerant cyanobacterium [27]. Further, the heat stress induced by seasonal variation in temperature increased MAAs production in certain algal species [27]. In contrast, cyanobacterium *Chlorogloepsis* when exposed to increased temperature or cold shock, downregulation of MAAs was observed, but when they were exposed to UV rays and salt stress, MAAs synthesis was induced [30]. Moreover, availability of nutrients like nitrogen can also influence MAAs production [28,31,32]. Thus, external stress factors like the spectral composition of light, nitrogen concentration, salinity of the culturing media and heat stress can impact the MAAs production in microalgae and cyanobacteria. Figure 1 represents the effect of various external abiotic factors on the production of MAAs in microalgae and cyanobacteria.

According to a review on MAAs published by Bhatia et al. (2011) [9], isolation and purification of MAAs from both algal and plant materials follow a common protocol. Crude drug samples of either plant or algal material are homogenized and the extraction of the sample was performed using methanol [9]. The general protocol for MAAs extraction and detection follows the use of ice-cold methanol combined with the application of HPLC and LC/MS [34]. High-performance liquid chromatography (HPLC) which is followed by characteristic UV wavelength detection was the most common method used for the quantification and detection of MAAs from the culture sample. However, as this procedure was found to be time consuming and less informative, liquid chromatography coupled with electrospray ionization mass spectrometry (LC/MS) is considered as a reliable method for MAAs detection and quantification today [35]. Vanessa Geraldes et al. in 2019, tested 69 native Brazilian cyanobacterial strains for the presence of MAAs accumulation using ultrahigh-performance liquid chromatography with diode array detection coupled with quadrupole time-of-flight mass spectrometry (UHPLC-DAD/QTOFMS) and identified nine different MAAs in 26 strains of cyanobacterial strains out of 69 strains tested. UHPLC-DAD/QTOFMS method was proven to be a fast and reliable method for the detection of MAAs in cyanobacteria as well as in algal strains [19]. Further, the Nuclear Magnetic Resonance (NMR), when coupled with LC/MS, showed capacity to accurately detect the MAAs’ chemical structures [24]. As a more advanced approach, liquid chromatography coupled with tandem mass spectroscopy (LC-MS/MS) was also introduced later which helped in rapid and accurate detection, and quantification of MAAs [36].

## 3. Structure and Properties of Mycosporine-like Amino Acids (MAAs)

**Figure 2:**
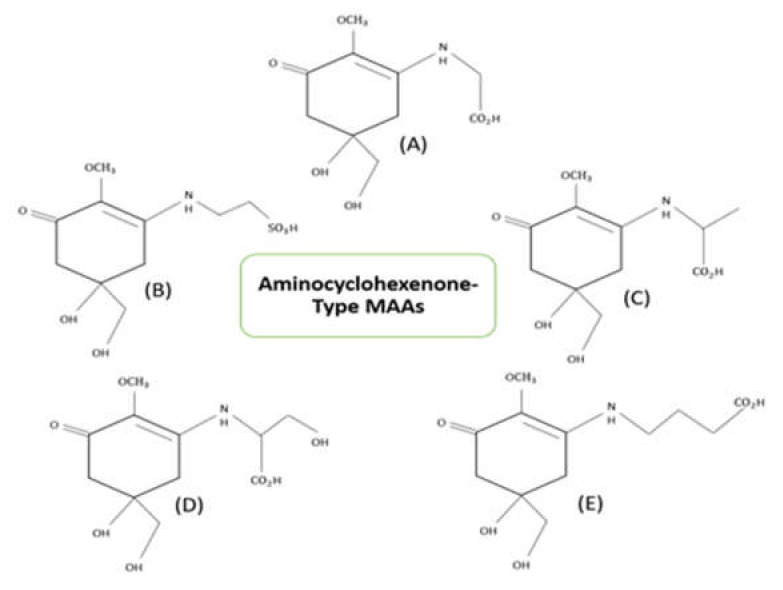
Structures of prominent Aminocyclohexenone-type MAAs (Monosubstituted MAAs) found in microalgae. Their absorption maximum in nanometer (nm) given in brackets. (**A**) Mycosporine-Glycine (310 nm), (**B**) Mycosporine-Taurine (309 nm), (**C**) Mycosporine-Alanine (310 nm) (**D**) Mycosporine-Serine (310 nm), (**E**) Mycosporine-γ-Aminobutyric Acid (310 nm) [3,27,40].

**Figure 3:**
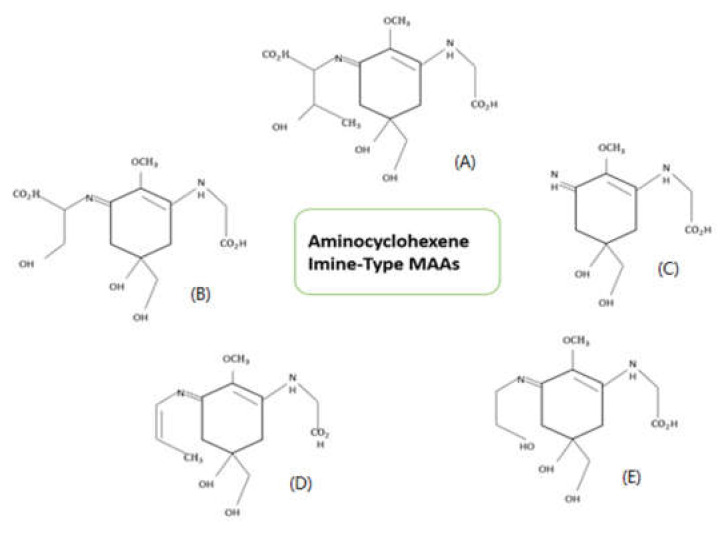
Structures of prominent Aminocyclohexene Imine-Type MAAs (Disubstituted MAAs) found in microalgae. Their absorption maximum in nanometer (nm) given in brackets. (**A**) Porphyra-334 (334 nm), (**B**) Shinorine (333–334 nm), (**C**) Palythine (320 nm), (**D**) Usujirene (357 nm) (**E**) Asterina-330 (330 nm) [3,27,41].

Mycosporine-like amino acids are multi-beneficial compounds with molecular weight less than 1.2 kDa [3]. These molecules are highly water-soluble when compared to other secondary metabolites like scytonemin. The zwitterionic form of MAAs absorbs UV radiation, with the utmost absorbance at 310–365 nm. The first finding of mycosporines P310 was done in terrestrial fungi *Stereum hirsutum*. The structure 1,2-methoxy-3-bis (hydroxymethyl) methylamino-5-hydroxy-5-hydroxymethyl-2-cyclohexen-1-one has been credited to mycosporine P310. Mycosporines were earlier depicted as differentiation or reproduction markers in fungal spores [37]. Nowadays, these compounds are well known for their multiple cellular functions such as osmo-protectant [10] and antioxidant [38] activities. Based on their core chromatophore structure, MAAs are classified into two types viz., cyclohexenone ring showing oxo-MAAs and cyclohexene imine ring showing imino-MAAs. Examples of oxo-MAAs are mycosporine-glycine and mycosporine-taurine, while imino-MAAs are shinorine, palythine, asterina-330, palythene, etc. [39]. Figure 2 and Figure 3 describes the structures of prominent MAAs belonging to these classes found in microalgae.

MAAs are denoted as microbial sunscreens which are natural and well known for its UV absorbing property [42,43,44,45,46]. UV rays are of three types UVA, UVB and UVC; among these UVA is absorbed strongly by MAAs. MAAs play supplementary roles, they can scavenge toxic oxygen radicals, in certain organisms. They are compatible solutes which are designed to protect against thermal, desiccation and salt stress. They also serve as an intracellular nitrogen reservoir in some of the organisms [11].

MAAs are mostly seen as solutes in cytosolic space, but in some terrestrial cyanobacterium, it was also reported as derivatives of mycosporines linked to amino acids and oligosaccharides secreted by their cells [47]. MAAs levels inside the cells are affected by certain environmental factors like nutrient availability, stress conditions, and UV. PAR (Photosynthetically Active Radiation), UVA and UVB radiation equally influences the production of MAAs inside the cells [48]. MAAs are promising metabolites in biotechnology because of their antioxidant properties and photostability over a wide range of temperature and pH. They can protect humans from immune suppression and photo-oxidative damage. MAAs are highly stable at room temperature and at a pH range of 4.5–8.5. However, the stability of the compound decreases with increase in temperature and pH. It has been observed that degradation of MAAs can happen much faster in alkaline conditions [49].

MAAs are highly stable because of their zwitterionic form, which is stabilized by resonance between equivalent structures and also due to its hydrogen bonds. Most of the MAAs have hydrogen bonded 3D structure with more than 12 bonds [50]. Extracellular oligosaccharides linked MAAs (OS-MAAs) have core ring structure linked to oligosaccharide side chains. Polysaccharides and external proteins are strongly attached to this side chain, which are present in some cyanobacterial species [47,51]. Based on the presence of attached protein, MAAs can be grouped into two forms, which are soluble non-protein associated MAAs present in algal invertebrate symbionts and protein associated MAAs present in asymbiotic metazoans [43].

### Properties

Mycosporines, have a common cyclohexenone ring system, which shares equal spectral characteristics and absorption maxima irrespective of their different molecular structures [46]. They are dependent on temperature and pH along with solvent nature rather than moieties linked to the ring structure. In the case of zwitterion, protonation occurs and delocalization of electrons can be prevented by ion pair electrons in nitrogen [52].

Further, the photoprotective role of MAAs is based on photostability, lack of fluorescence and radical production [53]. Anoxic conditions can increase the photostability. Mycosporine-like amino acids does not undergo any degradation under anaerobic conditions when treated with methanolic solutions. High degree of photostability also depends on the acidity of the medium, which was proved when structurally related keto-enolic gadusol was treated in aqueous solution for photostability studies [54].

The mycosporine-glycine is not stable under aerobic conditions and they have to be converted to methyl esters. In acidic media many MAAs are converted to methyl esters with HCl methanol treatment [55]. Temperature can also affect the stability of MAAs. However, even at high temperature absorption property of the compounds are not affected [56].

## 4. Biosynthesis Pathways of MAAs and its Derivatives

MAAs synthesis starts from an intermediate point of two different pathways, shikimic acid pathway and pentose phosphate pathway (Figure 1). It is believed that MAAs are mainly produced via shikimate pathway, otherwise called aromatic acid synthesis pathway. Shikimic acid pathway was studied multiple times to understand its role to produce 4-deoxygadusol, the precursor for MAAs production. It was first found in fungi *Trichothecium roseum* [57]. Radiolabeling experiments on *Chlorogloeopsis* confirmed shikimic acid pathway for MAAs production. The presence of exogenous tyrosine can act as the repressor and glyphosate can act as the inhibitor of shikimic acid pathway [58,59].This pathway transforms the precursor 3-dehyroquinate into 4-deoxygadusol via gadusol [58]. 

The pentose phosphate pathway is not the major pathway involved in MAAs production [60]. This was confirmed by the minimal production of shinorine, in the presence of shikimate pathway inhibitors. Shikimate pathway and pentose phosphate pathway are linked to each other (Figure 1). Their intermediates SH-7P (sedoheptulose 7-phosphate) from pentose phosphate pathway can be easily converted to erythrose 4-phophate, an intermediate in shikimate pathway, by trans-aldolase enzyme [61].

Monosubstituted cyclohexenones are formed by conjugation of glycine molecules and this forms the common intermediate in production of disubstituted imine type MAAs. It is said that amino acids are modified to form MAAs by condensation, dehydration, hydroxylation and decarboxylation. MAA sulphates and glycolates are also formed by adding required side chains to it [3].

Biosynthesis of essential amino acids in most of the organisms is via shikimate pathway. This pathway is present in algae, fungi, bacteria etc., but not in animals. They acquire MAAs through food chain [59,62]. Each species has different biosynthetic pathways for MAAs production and these pathways depend on abiotic factors like desiccation, temperature, nutrient availability, salinity etc., [63]. Spectral distribution and intensity of solar radiation are other factors which control MAAs biosynthesis [64].

## 5. Approaches to Increase MAAs Production in Microalgae

Microalgal species are good sources of MAAs. MAAs are utilized in various cosmetic and pharmaceutical industries and application of strategies such as genetic engineering can greatly enhance the productivity of MAAs in microalgae. Further, the transcriptomic studies to explore different genes involved in the biosynthesis of MAAs and its alternate pathways can help in these experiments.

Changing/applying various external parameters can have great influence on the expression of bioactive molecules like MAAs in microalgae. Along with the modification of cultural conditions, improving the metabolic pathways can increase the yield of desired metabolites. Thus, the identification of the genes responsible for the MAAs biosynthesis is crucial for future genetic engineering applications. However, possible consequences and environmental risks that can happen as a result of such genetically engineered organisms’ usage should be considered beforehand and their impact should be estimated [65,66].

Some microalgae like *Nannochloropsis*, *Chlamydomonas*, *Haematococcus*, *Dunaliella*, and *Phaeodactylum* are proven to be capable of nuclear transformation which make them suitable species for the application of genetic engineering. The use of proper codons, strong endogenous promoters, and insertion of 5′, 3′ and intron sequences, which are species specific can impact their expression stability. Thus, access to the microalgal genetic sequences have great importance in genetic engineering experiments. Biolistic or electroporation techniques are mainly utilized for the transformation of genes into microalgal cells and the transformants are selected using appropriate selection markers. Generating knockdown lines using RNA silencing technique is another approach for target gene deletion in microalgae along with homologous recombination [65,67]. Recent developments in CRISPR-Cas9 genome editing tool allow the deletion/knockdown of specific genes in eukaryotic cells. However, low efficiency due to off-target DNA cleavage and toxicity caused by vector-driven Cas9 nuclease activities are main limitations for their use in microalgal cells although direct delivery of Cas9 RNPs can improve the target efficiency in Chlamydomonas species [65,68]. However, the efficiency of different genetic engineering tools can be different in different microalgal species.

In cyanobacteria, MAAs biosynthesis involves a four-enzyme pathway [34,69]. However, great genetic variability was reported among different cyanobacterial species, in mys genes cluster, that are involved in the MAAs biosynthesis. The main enzymes, of mys cluster, NRPS and D-Ala-D-Ala ligase enzymes, encoded by mys-E and mys-D were found to be variable (present/absent) among these species. These findings by Rosic et al. [34] clearly indicate the involvement of certain other enzymes in MAAs synthesis pathway. Therefore, better understanding of their biosynthesis pathways and regulatory mechanisms is essential, and it is possible by various bioinformatic tools such as NGS sequencing and omics techniques [27,34,69].

In one of the studies, two cyanobacterial strains of *Scytonema* cf. *crispum* (UCFS10 and UCFS15) were used and putative mys gene clusters (mys ABC), important for mycosporine-glycine biosynthesis were identified [22]. The heterologous expression of mysABCE and the dehydrogenase gene in *Escherichia coli* led to the exclusive production of shinorine, which confirmed that *Anabaena* type mys cluster is responsible for the shinorine production in these species (confirmed by bioinformatic analysis). They were able to overexpress the mysABCE gene in *E. coli*, which resulted in more shinorine production [22].

## 6. Mycosporine-like Amino Acids: Applications and Uses

As MAAs are known to be multifunctional compounds, we tried to list some of the most prominent applications and uses of different types of MAAs.

### 6.1. MAAs as Antioxidants

Natural or artificial compounds that can prevent damage caused by free radicals are referred as antioxidants or free radical scavengers. The first antioxidant activity of MAAs were reported in mycosporine-glycine in 2004 [11]. High temperature exposure to a coral species; *Stylophora pistillata*, induced high catalytic activity, and high amount of superoxide dismutase was produced while disrupting photosynthesis machinery. Intracellular mycosporine-glycine amounts were decreased because of oxidative stress although MAAs other than mycosporine-glycine were not affected. Precursors of MAAs; 4-deoxygadusol also have a very strong antioxidant property. Imino-mycosporine compounds like shinorine, porphyra-334, asterine-330 cannot be oxidized [38,42,43]. Studies on the consequences of oxygen induced by illustration of eosine Y or methylene blue on lipid peroxidation, hemolysis of erythrocytes shows the efficiency of mycosporine-glycine in protecting biological systems [21].

Energy metabolism and production requires oxidation and it is linked to ROS production which serves as cell signaling molecule, triggering cellular processes such as cell division, inflammation and stress responses [70]. Oxidative stress gives rise to ROS which are commonly produced when more amount of energy is absorbed by photosystems. Hydroxyl radicals, singlet oxygen, hydroperoxyl radicals and superoxide anions are various kind of ROS [11]. In vitro analysis with DPPH (2,2-diphenyl-1-picrylhydrazyl) shows that mycosporine-2-glycine or mycosporine-glycine have more antioxidant property than shinorine and porphyra-334. Further in vitro studies using human melanoma cell lines or normal human skin fibroblast showed that mycosporine-glycine can prevent oxidative stress related cell death. This protective mechanism is through inhibitory effect of NF-kB signaling by mycosporine-glycine [71]. Among currently known MAAs, antioxidant activity is prominently shown by some of the mono-substituted, di-substituted and glycosylated MAAs.

### 6.2. UV Absorbing Compounds

Harmful human activities can cause ozone depletion and influx of UV radiation to earth. UVA and UVB reaches the ground level but UVC radiations are absorbed by the atmosphere. Strict photosynthetic active radiation requirement may increase the UV exposure, which can be detrimental to some organisms. Most of the highly energetic UV reaching the surface of earth can be absorbed by MAAs. They absorb UV radiations and release energy in the form of heat without producing ROS.

Melanin is a natural skin protection pigment placed above the nucleus and takes part in UV absorption and also acts as an antioxidant. It plays a key role in protecting keratinocyte cells against UV radiation. However, when exposure to UV radiation is excessive, an extra protection using topical applications such as sunscreen is required. These sunscreen compounds can be inorganic or organic but most of them have bad effects on the skin health. MAAs are the best choice of sunscreen which are compatible with biological systems [48]

### 6.3. Cell Viability/Proliferation

Shinorine, porphyra-334 and mycosporine-glycine doesn’t show any toxicity in murine fibroblasts, which was confirmed by second longer term direct incubation assay in the same cell line [72]. These MAAs were not showing any kind of toxicity in human TIG-114 fibroblast cells for 48 hours (concentration between 0–100 µM), but they gradually increased cell proliferation [73]. These MAAs have shown perfect blend in wound healing property and cell proliferation.

### 6.4. NRF-2 Activation

The Keap1 (Kelch-like ECH-associated protein 1) - Nrf2 (erythroid 2–related factor 2 protein) pathway is an important regulator of oxidative stress in cells. Reactive oxygen species (ROS) are recognized by the Keap1, which leads to the release of Nrf2, and this Nrf2 is involved in the activation of oxidative stress related cytoprotective genes [74]. MAAs like porphyra- 334 and shinorine were found to have a competitive interaction with the Keap1 protein at the Nrf2 binding site under UVR exposure. These MAAs acted as antagonists to the Keap1-Nrf2 binding, which increased the Nrf2 release, and upregulated cytoprotective gene activation under oxidative stress conditions. Thus, the MAAs shinorine and porphyra-334 act as the activators of the cytoprotective Keap1-Nrf2 pathway under UV induced stress conditions [41].

### 6.5. DNA Damage/Erythema

Genomic mutations by the production of DNA photoproducts are one of the most serious damaging effects of UV rays’ exposure [75]. Prolonged exposure to UV-B rays can cause erythema/sunburn, DNA damage and immunosuppression. A mycosporine called Collemin-A totally inhibited sunburn, when it was applied on the skin before the UV-B exposure. These compounds blocked the pyrimidine dimer formation in human skin cells. It was also found that high MAAs concentration in the cells of certain dinoflagellates reduced the UV induced damage and, inhibition of motility and photosynthesis in them [76].

### 6.6. Inflammation

Overexpression of biomarkers of skin leads to conditions like psoriasis. Topical application of mycosporine-glycine, shinorine and porphyra-334 are studied against these conditions and it was found that these can be controlled with high concentration of mycosporine-glycine and low concentrations of shinorine [21].

### 6.7. Photoaging

Long term UV rays’ exposure leads to wrinkles and sagging in skin where matrix metalloproteinases are activated [77]. These metalloproteinases can be inhibited by porphyra-334 which reduces elastase activity dose dependently [78]. 

### 6.8. MAAs as Anticancer Agents

Antiproliferative activities on neoplastic cells put MAAs into the group of anticancer agents. Murine skin melanoma cells when administered with red algal extracts rich in various MAAs like shinorine, porphyra-334, etc. showed dose dependent inhibition of cell proliferation [20]. Presence of additional MAAs led to greater antiproliferative activity in murine melanoma cells [79]. Porphyra type MAAs were able to block UV induced production of DNA lesions like cyclobutane pyrimidine dimers (CPDs) and pyrimidine-(6-4)-pyrimidone photoproducts (6-4-PP), which helped to protect DNA molecules in *P. yezoensis* cells [80]. 

### 6.9. MAAs as Wound Healing Agents

Wound healing proceeds in an organized way, which includes homeostasis, where wounds clot, constrict to resist blood flow and platelets stick to seal the cut part. Inflammation is the other phase in which localized swelling occurs. Proliferative phase and maturation phase are where rebuilding and remodeling occur respectively. These processes require many factors including epidermal growth factors. MAAs were also reported to promote wound healing in HaCaT human keratinocytes cells. MAAs stimulates the activation of tooting pathway of focal adhesion kinases (FAK) and mitogen activated protein kinases (MAPK). When MAAs are administered FAK phosphorylation occurs, which, in turn, facilitates the activation of MAPK’s extracellular signal regulated kinase [8].

## 7. Summary and Future Prospects

In this review, we summarized all the important properties, structure and applications of MAAs. Due to their high photoprotective and antioxidant activities, they are being utilized in pharmaceutical and cosmetic industries nowadays. Unfortunately, utilization of their biological sources for the mass production of these economically important molecules has not been explored very well. Thus, this review focused mainly on microalgae as a potential biological source of MAAs and, the prospects and challenges involved in their genetic manipulation for the increased MAAs expression.

Microalgae are microscopic algal species, which are known to be a good source of various bioproducts. As they are easily culturable and require less generation time, microalgae are considered to be a good option for the production of mycosporine-like amino acids. As microalgae are unicellular, photosynthetic organisms, they can reduce the environmental pollution to some extent, increasing the quality of air we breathe. This reassures the fact that microalgae are one of the best sources for MAAs production.

In microalgae, extraction of MAAs using solvent, which only consists of water and a volatile additive like methanol, followed by their detection and quantification using HPLC or LC/MS is the common procedure used. This resulted in the detection of great diversity in the occurrence of different MAAs in various microalgal species. Combination of HPLC and LC/MS or NMR and LC/MS are some common protocols followed today to increase the detection and quantification efficiency of MAAs.

In order to make microalgal species capable of producing large amounts of MAAs, certain improvements in the culture conditions or external parameters can be utilized. Modifying the temperature, salinity, nitrogen concentration and UV/fluorescence exposure during the culturing of microalgae in such a way that they are exposed to stressful environment can induce the overexpression of MAAs in them. However, as these are stressful conditions for the microalgae, they can lower their cell division, decreasing thus the overall productivity of the biomolecules.

Application of biotechnology in the production of MAAs from microalgae at an industrial level is the most promising future prospect in the case of these biologically important molecules. Identification of the genes and regulatory mechanisms involved in MAAs biosynthesis in various microalgal species, using certain bioinformatics tools and multiple omics approaches is important for this purpose, as only limited information regarding the genomic sequences of microalgal species are currently available. Various genetic engineering techniques can be utilized for the manipulation of these genes, particularly mys cluster of genes, involved in MAAs biosynthesis in certain microalgal species. Heterologous cloning techniques are utilized for the overexpression of these genes for high production of certain MAAs like shinorine. Molecular cloning techniques and, genome editing tools like zinc-finger nucleases (ZFNs), transcription activator-like effector nucleases (TALEN), and clustered regularly interspaced short palindromic repeats (CRISPR/Cas9) can be great tools in the genetic manipulation (knock-down or knock-in) of these genes in microalgae. However, low efficiency, cytotoxicity and restricted knowledge in the genomic information of microalgal species are the main limitations in these applications. Techniques like CRISPR-Cas9 have great scope in future for the mass production of MAAs from microalgae because of its simplicity and accuracy, if the limitations associated with their application in them are solved. Genome editing in high biomass producing microalgae for the overexpression of MAAs biosynthesis genes (enhancing their promoters and pathway regulation strategies) or genetic manipulation in high MAAs producing microalgal species to increase their biomass production (increasing photosynthetic efficiency) are two alternative routes in which genetic engineering can be carried out for the increased MAAs production in microalgae. Genetic engineering of the organisms like microalgae for the mass production of MAAs can bring great future prospects and applications for this high valuable compound. Thus, the challenges involved in the culturing and genetic engineering of the microalgae for the industrial production of MAAs need to be addressed very well in the future.

## Figures and Tables

**Table 1:** Mycosporine-like amino acids (MAAs) produced by various microalgal/cyanobacterial strains and their applications.

Sl.No.	Type of MAA	Microalgal/Cyanobacterial Source	Applications	Reference
1.	7-O-(β-arabinopyranosyl)-porphyra-334	*Nostoc commune*	Antioxidant, Sunscreens	[12,13]
2.	Hexose-bound porphyra-334 and its derivative	*Nostoc commune*	Sunscreens, antioxidant, Chemotaxonomic marker of *N. commune* genotypes	[12,13,14]
3.	Porphyra-334	*Nostoc commune*, *Chlamydomonas hedleyi*, *Alexandrium* sp. (*A. catenella*, *A. minutum*, *A. tamarense*, *A. excavatum*), *Gloeodinium viscum*, *Gymnodinium catenatum*, *Gyrodinium dorsum*, *Microcystis aeruginosa*, *Scytonema* sp., *Acetabularia mediterranea*, *Lyngbya* sp., *Aphanothece halophytica*	Sunscreens, Cosmetic creams (anti-ageing), Wound healing agent, Anti-inflammatory agent, Anti-cancer agent	[3,7,8,15,16,17,18,19,20,21]
4.	Two hexose-bound palythine-threonine derivatives	*Nostoc commune*	Sunscreens, Antioxidant, Chemotaxonomic marker of N. commune genotypes	[12,13,14]
5.	Mycosporine-γ-Aminobutyric Acid	*Nostoc commune*	Pharmaceutical products - have anti-tumor properties and helps in lowering blood sugar level and hypertension.	[12,13,14]
6.	Shinorine	*Chlamydomonas hedleyi*, *Alexandrium* sp. (*A. catenella*, *A. minutum*, *A. tamarense*, *A. excavatum*), *Gymnodinium catenatum*, *Gyrodinium dorsum*, *Scytonema* cf. *crispum*, *Microcystis aeruginosa*, *Scytonema* sp., *Calothrix* sp., *Aphanothece halophytica*, *Acetabularia mediterranea*, *Lyngbya* sp.	Wound healing agent, sunscreens and cosmetics, anti-inflammatory agent, antioxidant, pharmaceutical products - cure for Psoriasis like conditions, anti-cancer agent	[7,15,16,19,20,21,22,23]
7.	Mycosporine-Glycine	*Chlamydomonas hedleyi*, *Alexandrium* sp. (*A. catenella*, *A. minutum*, *A. tamarense*, *A. excavatum*), *Gloeodinium viscum*, *Gymnodinium catenatum*, *Calothrix* sp., *Aphanothece halophytica*	Wound healing agent, sunscreens and cosmetics, anti-inflammatoryagent, pharmaceutical products -cure for Psoriasis like conditions	[16,19,21,24]
8.	Palythine	*Alexandrium* sp. (*A. catenella*, *A. minutum*, *A. tamarense*, *A. excavatum*), *Acetabularia mediterranea*, *Lyngbya* sp., *Pseudococcomyxa* sp., *Gyrodinium dorsum*	Sunscreens and cosmetics, antioxidant	[7,16,19,23,25]
10.	Asterina-330	*Lyngbya* sp., *Alexandrium* sp. (*A. catenella*, *A. minutum*, *A. tamarense*, *A. excavatum*)	Antioxidant	[7,16,23]
11.	Mycosporine-taurine	*Synechocystis* sp.	Antioxidant, sunscreens	[26]
12.	Dehydroxylusujirene	*Synechocystis* sp.	UVA filter, antioxidant	[26]

## Data Availability

Not applicable. Data is utilized from published articles, which are available in the public domain.
